# Editorial: Human milk, nutrition and infant development

**DOI:** 10.3389/fnut.2024.1525112

**Published:** 2024-12-02

**Authors:** Francisco J. Pérez-Cano, Veronique Demers-Mathieu, Claude Billeaud

**Affiliations:** ^1^Physiology Section, Department of Biochemistry and Physiology, Faculty of Pharmacy and Food Science, University of Barcelona (UB), Barcelona, Spain; ^2^Institute of Research in Nutrition and Food Safety (INSA), UB, Barcelona, Spain; ^3^Janssen Pharmaceutical Companies of Johnson & Johnson, San Diego, CA, United States; ^4^Centre Hospitalier Universitaire de Bordeaux, Bordeaux, France; ^5^European Association for Paediatrics Education (AEEP/EAPE), Bordeaux, France

**Keywords:** breast milk, immunoglobulins, PUFA, metabolites, oligosaccharides, milk banks

Breast milk is the gold standard for infant nutrition and feeding. Human milk is composed of a thousand components with nutritional functions and some of them as additionally bioactive properties. Breast milk composition differs between mothers due to maternal background, immunity, nutrition, lifestyle, and other confounding factors. In addition, the same mother's milk composition varies over time; colostrum contains the highest level of active proteins compared to transitional and mature breast milk to provide maximal immunity to the newborn. Indeed, the levels of bioactive proteins and macronutrients are higher in preterm milk than in full-term milk to promote their development and compensate for their immaturity. Breast milk composition is also affected by the mother's diet. Although milk proteins and carbohydrates are only slightly influenced, there is a strong correlation between dietary lipids and breast milk, as well as minerals, vitamins, and trace elements. Besides these factors, other situations, globally considered as the “exposome,” can have also an influence on human milk composition. The discovery of new components in the mother's milk remains challenging due to the determination of the specific and accurate roles in infant growth and protection. The composition of human milk is even more complex since the evolution of different metabolomic techniques, which provide a very complete and up-to-date nutrient-by-nutrient record of breast milk.

This Research Topic compile compendium of 26 up-to-date original research and review articles focused on several properties of breast milk, including nutrition, immunity, and child development. In addition, some articles describe the implications of incorporating constituents to design breast milk substitutes as well as the use of donor human milk to compensate for the lack of breast milk/breastfeeding, especially for vulnerable premature neonates.

First of all, three interesting articles highlight the importance of breastfeeding and how their practices can vary widely across different cultures, with traditions and beliefs. These distinct cultures lead to shaping how, when, and for how long mothers breastfeed their infants. Whereas, the research done by Rodrigues et al. shows how relevant are the demographic, health, and economic factors in the feeding practices in three Latin American countries in three decades, Negesse et al. describes the influence of living or not in a secure area, among other factors, on the exclusive breastfeeding practice. Finally, the relationship between breastfeeding duration and Body Mass Index (BMI) has been the subject of several studies, particularly in relation to maternal and child health. Sun et al. describe in this Research Topic their findings in a 9-year population-based study that highlight the importance of breastfeeding to reduce childhood overweight/obesity and prevent diseases, both in clinical and public health settings.

Another important group of articles summarizes the wide variety of bioactive components present in breast milk and their role in both maternal and infant health.

Fat in human milk primarily consists of triglycerides, which make up about 98% of the total fat content and are forming the Fat Globule Core. The milk fat globule membrane (MFGM) contributes to 0.2–2% of total fat in breast milk and has evidenced potential benefits for brain development. For that reason, Zhang et al. studied its effects in piglets, demonstrating its role in enhancing the connection of white matter fiber trace and improving neonatal piglets' learning and memory abilities. In this subject but at human level, another article within this Research Topic describes a randomized, double-blind, controlled clinical trial that showed that the combination of phospholipids and long chain polyunsaturated fatty acids supports neurodevelopmental outcomes in infants (Ren et al.). The fat necessity for this and other effects has led to the development and large-scale production of human milk fat analogs, as is the case of the study performed by Zhou et al. based on fermentation of microalgae. In addition, in breast milk, as in other natural sources, unsaturated fatty acids can have either cis or *trans* configuration double bond in their chain with distinct physiological effects. Hatem et al. revise in their article that trans isomeric fatty acids in human milk could impact infant health and development; but since WHO rules and improvement of Hydrogenated oils the *TFAs* human milk levels is < 1% of fatty acids, insuring safety and positive outcome. Overall, the relationship between fat milk composition and infant's development is guaranteed. Omega 3 and particularly EPA, DHA reduces inflammatory, obesity, cancers, but also infant neurodevelopment and visual function. Maternal docosahexaenoic acid supplementation during lactation improves exercise performance, enhances intestinal glucose absorption and modulates gut microbiota in weaning offspring mice (Lu et al.). These results are in line with several longitudinal studies on pre-gestational, gestational, and lactation periods in which it has been observed that the maternal fish and omega 3 consumption influence the outcomes in 17 years old infants. The omega 3 maternal intake decreases CpG sites numbers on DNA methylation and the biological age, so improves the infant and adult programing outcome.

After fat and lactose, human milk oligosaccharides (HMO) are the most abundant solid component in human milk and their composition varies during lactation and exert a broad list of effects on the infant's health. Not all mothers have same HMO composition or are functionally the same. However, Mainardi et al. showed how the different HMO could act synergistically in influencing infant's growth. 2′-FL concentration cut off of 200 mg/L separated secretors and non-secretors with 100% accuracy: < 200 mg/L in the milk of non-secretor women (see article Liu et al.), while it is the most abundant HMO in the milk of secretors. Two articles in this compendium bring evidences of their effects. On the one hand, it is showed the influence of microbially fermented 2'-fucosyllactose on neuronal-like cell activity in an *in vitro* co-culture system (Kuntz et al.), and also its effects in influencing systemic immune development and function in piglets when it is combined with *Bifidobacterium longum* subspecies infantis (Daniels et al.). Overall, HMO, as undigestible sugars who are necessary to reach the gut intact, are able to influence the gut microbiome, and at the same time some factors influence its HMO composition, such as maternal geographic, environment, genetics, childbirth (caesarian vs. vaginal), antibiotics treatment but also the maternak diet and microbiome (Ajeeb et al.).

In the last years, the presence of human milk extracellular vesicles (HM-EVs) have been also a matter of study. These are lipid bilayer membrane vesicles (50–200 nm) containing myriad signaling molecules including proteins, lipids, microRNAs, mRNAs, and other biomolecules protected from degradation. Gómez-Ferrer et al. demonstrated in their study that the omega-3 oxylipins in HM-EVs can have pro-resolutive actions in gastrointestinal inflammation. In addition, another study showed the associations between the miRNAs present in HM-EVs and oligosaccharide concentrations in human milk (Holzhausen et al.).

Globally, breast milk is known to modulate infant intestinal gut barrier. A comparative analysis between raw and pasteurized breast milk was conducted by Rodríguez-Camejo et al. to understand the adverse effects of heat-treatment on cellular functions associated with the gut epithelial barrier and responses to inflammatory stimuli. These authors reveal that all milk types stimulated epithelial cell proliferation, but raw colostrum increased cell migration and interfered with the attachment of *E. coli* on epithelial cells.

In addition, components from other milk species have shown bioactivity comparable to that from human origin. As example, an expert panel reviewed the safety data and physiological role of dietary bovine osteopontin in infancy. They concluded that osteopontins from human milk and bovine milk had comparable plasma absorption and properties on cognition and immunity (Fleming et al.).

As mentioned before, human milk is a dynamic fluid which composition changes due to many internal and external factor such as the lactational period, geography, diet, and environment. For instance, it is shown that lactational and geographical aspects have an influence on the variation in the concentration of six oligosaccharides in Chinese breast milk. These results, from a multicenter study over 13 months postpartum developed by Liu et al. demonstrated that, compared with other studies, the variation among different geographical sites within China is smaller than the variation observed between different countries. Specifically, it is showed that that 79% of Chinese mothers have the secretor phenotype and 21% express a non-secretor phenotype respectively with high or low 2′FL level in the human milk; conversely, 3′-FL was ubiquitous and increased as in others countries all over the lactation. Human milk microbiome, the microogranisms who can also use these oligosaccharides, also differs depending on several factors, and it seems to be associated with maternal diet and infant growth, among others (Ajeeb et al.).

On the other hand, the breast milk immune composition also varies during the lactation period. The transition stage of lactation, which is the short period of days between colostrum and mature milk, is the one with less information regarding its immunoglobulin composition or immunoglobulinome. The article of Rio-Aige et al. characterizes two different clusters of milk types in basis of immunoglobulins and cytokines presente from the first to the last day of transitional milk, which have been called immunotypes. For that 75 mother-child pairs from the MAMI cohort. Following with the immune composition of human milk, it has been also a matter of study during the COVID-19 pandemic. It has been demonstrated the presence of specific Ig against the SARS-CoV-2 both after infection and vaccination. In their article, de Graaf et al. explores the human milk polyclonal IgA1 response to repeated SARS-CoV-2 vaccinations by using LC–MS based fab profiling.

Maternal factors also can influence the milk hormone concentrations, as is explored in the systematic review by Qureshi et al.. The revised studies consistently revealed the presence and varying concentrations of adiponectin, leptin, insulin, cortisol, and ghrelin in breast milk. Some of them change in relation to maternal factors such as BMI, weight and other health indicators (environment, lifestyle, and smoking). To date, maternal pregestational BMI increases milk leptin level and maternal diabetes increases milk insulin levels and increase also fat body mass of their infants. For the others hormones there is no evidence link between their milk content and maternal factors.

Prematurity is defined as a birth that occurs before 37 completed weeks of gestation, resulting in infants who may face a higher risk of health complications and developmental challenges due to their early arrival. Breast milk in this early period could have a critical role. This Research Topic, includes an article by Pütz et al. showing the association of different types of human milk with bronchopulmonary dysplasia in preterm infants. A lower prevalence of bronchopulmonary dysplasia was found in neonates fed with mother's own milk compared to those fed with donor human milk as well as when breast milk was used “fresh” compared to “frozen/thawed.” These results underline the importance of mother's own milk and its proportion administered on the prevention of bronchopulmonary dysplasia. In addition, human milk needs to be fortified in some situations, as in low birth weight neonates, such as prematures. Biasini et al. reported the long-term advantages of protein-fortified human milk in this population. Specifically, the article shows that neonatal enteral protein supplementation in very low birth weight preterm infants leads to no positive nor adverse consequences in long-term assessment, suggesting that benefits are restricted to the neonatal term and 1st years of age.

In the absence of the mother's own milk, Donor Human Milk (DHM) is essential for the nutrition of premature babies. Human milk banks (HMB) are essential for collecting, processing, storing, and redistributing DHM. There are around 900 HMBs around the world, unevenly distributed, including 250 in Europe and many in South and North America. The creation of an HMB in a developing country is crucial and poses problems of organization and funding, as it is shared in the article of Nakibuuka et al. which explains the setting up of the first human milk bank in Uganda. Its novelty contraposes to the HMB of Vietnam (Thi Tran et al.), which has been in existence for 5 years, collects 10,000 L of milk in 5 years and analyses the factors influencing the volume of donations (14 L/donor), but also faces the financial difficulties for poor people ($60/L).

A critical aspect for the HMB is to ensure the preservation quality of the components of raw breast milk and its microbiological safety. For that reason, although pasteurization (Holder) is the most used technique for ensuring preservation of DHM, new alternatives need to be implemented to better preserve its quality (Moro et al.).

In summary, the Research Topic includes interesting articles focusing on the human breast milk composition in terms of nutrients, biological compounds, microbiome, HMO, and extracellular vesicles, among others. All of them participate boosting the immune and metabolic system development and support the short and long-term health of newborns. The critical aspects of maternal exposome in milk composition and the milk banks function is also of importance in this Research Topic.

Further studies are encouraged to expand the knowledge of breast milk and complete this fist volume, especially those focused on proteins, their role in nutrition and their bioactive properties in immunity, the microbiome and neurodevelopment; minerals (phosphorus-calcium metabolism), trace elements and vitamins and the different supplements to be administered to the mother before, during gestation and during lactation; antioxidants in breast milk such as phenolic components; maternal factors in the perinatal environment (toxins, pollutants, exercise, smoking, alcohol, over- or under-nutrition, pathologies, and nutrition) that can affect the medium- and long-term outcome of the child. Finally, research into the genetic and epigenetic regulation of the volume and composition of breast milk, metabolism and growth of the fetus, newborn, and child should be analyzed in depth.

